# Temperature effects on the contractile performance and efficiency of oxidative muscle from a eurythermal versus a stenothermal salmonid

**DOI:** 10.1242/jeb.242487

**Published:** 2021-08-05

**Authors:** A. Kurt Gamperl, Douglas A. Syme

**Affiliations:** 1 Department of Ocean Sciences, Memorial University of Newfoundland, St John's, NL, CanadaA1C 5S7; 2 Department of Biological Sciences, University of Calgary, Calgary, AB, CanadaT2N 1N4

**Keywords:** Thermal tolerance, Heart, Myocardium, Red muscle, Acute, Temperature acclimation, Muscle performance, Muscle efficiency

## Abstract

We compared the thermal sensitivity of oxidative muscle function between the eurythermal Atlantic salmon (*Salmo salar*) and the more stenothermal Arctic char (*Salvelinus alpinus*; which prefers cooler waters). Power output was measured in red skeletal muscle strips and myocardial trabeculae, and efficiency (net work/energy consumed) was measured for trabeculae, from cold (6°C) and warm (15°C) acclimated fish at temperatures from 2 to 26°C. The mass-specific net power produced by char red muscle was greater than in salmon, by 2-to 5-fold depending on test temperature. Net power first increased, then decreased, when the red muscle of 6°C-acclimated char was exposed to increasing temperature. Acclimation to 15°C significantly impaired mass-specific power in char (by ∼40–50%) from 2 to 15°C, but lessened its relative decrease between 15 and 26°C. In contrast, maximal net power increased, and then plateaued, with increasing temperature in salmon from both acclimation groups. Increasing test temperature resulted in a ∼3- to 5-fold increase in maximal net power produced by ventricular trabeculae in all groups, and this effect was not influenced by acclimation temperature. Nonetheless, lengthening power was higher in trabeculae from warm-acclimated char, and char trabeculae could not contract as fast as those from salmon. Finally, the efficiency of myocardial net work was approximately 2-fold greater in 15°C-acclimated salmon than char (∼15 versus 7%), and highest at 20°C in salmon. This study provides several mechanistic explanations as to their inter-specific difference in upper thermal tolerance, and potentially why southern char populations are being negatively impacted by climate change.

## Abstract (precis)

**Summary:** Additional mechanistic insights indicate why Atlantic salmon are more tolerant of high temperatures than Arctic char, and why char are particularly vulnerable to climate change.

## INTRODUCTION

Muscle contractile performance is critical to many aspects of fish biology, including cardiovascular function, swimming, migration and foraging, and dominates the energy used by active fishes (Gerry and Ellerby, 2014). Hence, impaired muscle performance, or marked changes in muscle efficiency, with changes in temperature could limit thermal tolerance and temperature-dependent performance of the whole animal (Wang and Overgaard, 2007; Eliason et al., 2011; Eliason and Anttila, 2017). Research on the effects of thermal acclimation on fish muscle performance has primarily focused on the effects of cooling and cold acclimation, and these studies report that cold acclimation improves contractile performance at low temperatures (reviewed by Johnston and Temple, 2002; Syme, 2006; Klaiman et al., 2014; James and Tallis, 2019). The effects of acclimation to higher temperatures, and warming to temperatures that approach the upper thermal limits of fish, on muscle function are not as well described, although recent concerns about accelerated climate warming and the increasing severity and frequency of heat waves (e.g. Clarke et al., 2007; Fey et al., 2015; Oliver et al., 2018; Frölicher et al., 2018; Stillman, 2019) have spurred interest in the effects of warming and how it may impact fish physiology, including effects on cardiac and skeletal muscle function (e.g. Klemetsen et al., 2003; Elliott and Elliott, 2010; Clark et al., 2011; Martins et al., 2012; Chen et al., 2013; Christen et al., 2018; Shuman and Coughlin, 2018; James and Tallis, 2019; MacMillan, 2019; Pichaud et al., 2019; Haverinen and Vornanen, 2020; Nudds et al., 2020).

Responses of fish to warming have mostly been studied at the level of phenotypic plasticity (Crozier and Hutchings, 2014), using relatively short generational or within-generation responses to warming (such as acclimation and rearing studies). However, there is some evidence of phenotypic plasticity and adaptations in fish physiology and muscle function following exposure to warm environments. Chen et al. (2015) reviewed studies that support increased tolerance to high temperatures in fishes exposed to warm environments, and provided evidence of increased tolerance to warming in rainbow trout (*Oncorhynchus mykiss*) over generations of exposure to a warm climate. Rodnick et al. (2004) report uncharacteristically high thermal tolerance in redband trout, a strain of rainbow trout that inhabits warm desert environments. Both Anttila et al. (2014) and Gerber et al. (2020) suggest that acclimating Atlantic salmon to 20°C [a temperature only 3°C below their maximum holding temperature (Hvas et al., 2017; Gamperl et al., 2020) and 6°C below their critical thermal maximum (CT_max_) (Penney et al., 2014; Leeuwis et al., 2019)] improves this species' temperature of heart failure and cardiac mitochondrial function at elevated temperatures. Finally, although very warm temperatures impair the power output of red skeletal muscle in Pacific bonito (*Sarda chiliensis*), this effect is not seen in red muscle of the regionally endothermic yellowfin tuna (*Thunnus albacares*) (Altringham and Block, 1997).

In contrast to these apparent evolutionary and phenotypic responses to warm temperatures, other studies suggest that fish muscle function may show little plasticity in response to warming. Sandblom et al. (2016) report no scope for changes in perch (*Perca fluviatilis*) heart function in response to warming. Muñoz et al. (2015) did not find a change in the temperature at which the heart becomes arrhythmic in juvenile Chinook salmon (*Oncorhynchus tshawytscha*) reared at 4°C warmer than current environmental temperatures. Franklin et al. (2013) found reduced scope for respiration and cardiac output with warming in species of sculpin that inhabit Arctic habitats versus those in more temperate regions. Further, Shuman and Coughlin (2018) and Coughlin et al. (2020) report that the myotomal (skeletal) muscle of rainbow trout has limited capacity/plasticity to acclimate to warmer temperatures.

The Arctic char [*Salvelinus alpinus* (Linnaeus 1758)] and Atlantic salmon (*Salmo salar* Linnaeus 1758) both exist as anadromous and land-locked populations in Canada and in Europe, and there is concern about their abilities to tolerate environmental warming (Klemetsen et al., 2003; Elliott and Elliott, 2010; Gilbert et al., 2020; Layton et al., 2021). The Arctic char has a holarctic distribution and generally lives in areas where average water temperatures rarely exceed 16°C, but maximum temperatures can reach ∼20°C for short periods during warm events (Gilbert et al., 2016, 2020). In contrast, Atlantic salmon are found in the Atlantic Ocean ranging from the northern USA and Spain to Greenland, and experience maximum summer water temperatures of 28–30°C in New Brunswick streams (Caissie et al., 2014). These differences in distribution and maximum water temperatures are reflected in aspects of their physiology and thermal biology. For example, the CT_max_ for adult Arctic char is 21–23°C (Elliott and Elliott, 2010; Penney et al., 2014; Christen et al., 2018; Gilbert et al., 2020), notably lower than that for Atlantic salmon, ∼26°C (Penney et al., 2014; Leeuwis et al., 2019). Further, these interspecific differences in upper thermal tolerance appear to be at least partially determined by cardiorespiratory capacity. While the rate of oxygen consumption (*Ṁ*_O_2__) and various measures of cardiovascular performance are not significantly different between these species when acclimated to 9.5°C, maximum *Ṁ*_O_2__ and aerobic scope are significantly lower (by 35–40%) in char, maximal heart rate (*f*_H_) is approximately 15% lower in char, and the cardiac mitochondria of char are not as efficient [i.e. they have higher State 2 and 4 respiration and lower respiratory control ratios (RCRs)] as compared with salmon (Penney et al., 2014). These differences likely limit the ability of char to tolerate warmer temperatures. However, we do not understand how acclimation temperature might influence these results, or have a comprehensive understanding of the mechanisms that underpin the differences in upper thermal tolerance and thermal-related physiology between these species (Elliott and Elliott, 2010). This is an interesting question given that the physiological basis(es) for differences in thermal tolerance are being vigorously debated (i.e. see Clark et al., 2013; Pörtner et al., 2017, 2018; Jutfelt et al., 2018; MacMillan, 2019; Ern, 2019; Haverinen and Vornanen, 2020; Lefevre et al., 2021; Andreassen, in revision), and that a more thorough understanding of the thermal biology of these species would assist in the formulation of policies, regulations and conservation measures, in particular to protect Arctic char at the southern distribution of their historical range.

To understand the more limited thermal tolerance of Arctic char as compared with Atlantic salmon, we tested several specific hypotheses: (1) that the red skeletal muscle and myocardium of adult salmon are better suited to function at warmer temperatures from a mechanical perspective; (2) that char may be capable of enhancing cardiac and skeletal muscle performance at warm temperatures through acclimation (phenotypic plasticity); and (3) that the energetic efficiency of cardiac contraction may be less in char than in salmon, particularly at warmer temperatures. To do this, we measured the effects of thermal acclimation (6 versus 15°C) and differences in test temperature (2–26°C) on the ability of red skeletal muscle and ventricular cardiac muscle from these species to produce maximal mechanical power across a range of contraction frequencies that encompass those that result in maximal net power output (i.e. 0.5–7 Hz for red muscle, and 20–140 contractions min^−1^ for myocardial trabeculae). Because net power is the sum of shortening and lengthening power, it is also informative to examine these parameters. Finally, we investigated the energetic efficiency of ventricular trabeculae from 15°C-acclimated fish at temperatures of 15, 20 and 25°C and at frequencies of 70–120 contractions min^−1^. This latter experiment was performed because the data of Penney et al. (2014) suggest that the char would have a lower efficiency (work output for a given amount of energy used) of myocardial contraction. A lower cardiac mitochondrial and myocardial efficiency in Arctic char may contribute to a reduced ability to tolerate warmer temperatures.

**List of symbols and abbreviations**

CT_max_: critical thermal maximum
*f*_H_: heart rate
*Ṁ*_O_2__: oxygen consumption rate
*P*_O_2__: oxygen partial pressure
RCR: respiratory control ratio
RVM: relative ventricular mass
*V̇*_O_2__: rate of oxygen uptake

## MATERIALS AND METHODS

All procedures were approved by the University of Calgary and Memorial University of Newfoundland (MUN) animal care committees, and followed Canadian Council on Animal Care guidelines. Atlantic salmon and Arctic char of mixed sex were obtained from aquaculture sea-cages in Bay D'Espoir, Newfoundland, Canada. The char were originally Labrador (Canada) stock, whereas the salmon were of Saint John River (New Brunswick, Canada) origin. The fish were held in approximately 6000-liter tanks at the Dr Joe Brown Aquatic Research Building of the Ocean Sciences Centre (MUN) for 4 months in seawater at either 6 or 15°C before experiments began. Temperatures of 6 and 15°C were chosen for study as streams inhabited by char in Nunavut during the summer did not drop below ∼7°C, and the mean daily temperature reported was approximately 15–16°C (Gilbert et al., 2016).

Char used in the study averaged 1.43±0.03 kg (mean±s.e.m.) in mass and 43.4±0.46 cm in fork length, and salmon averaged 1.61±0.06 kg in mass and 47.8±0.50 cm in fork length; see Tables 1 and 2 for details about the fish used in specific experiments. Water oxygen levels were always greater than 95% air saturation, the photoperiod was 12 h:12 h light:dark, and the fish were fed a commercial salmon diet at a ration of 1% of body mass every second day.

**Table 1.**
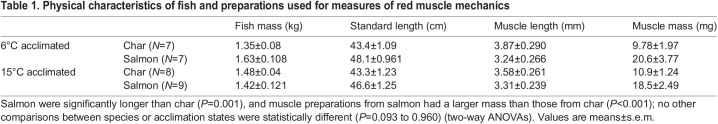
Physical characteristics of fish and preparations used for measures of red muscle mechanics

**Table 2.**
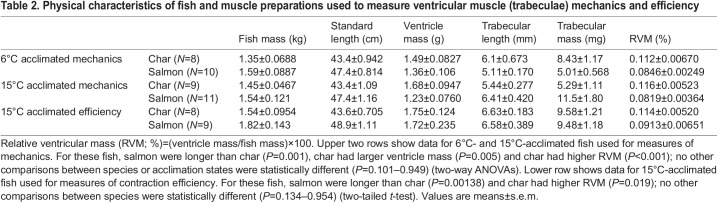
Physical characteristics of fish and muscle preparations used to measure ventricular muscle (trabeculae) mechanics and efficiency

### Muscle mechanics

For experiments using red skeletal muscle, procedures were similar to those described in Syme et al. (2008). Muscle preparations were isolated from the red muscle along the lateral line near the posterior margin of the dorsal fin. They were then mounted into the same apparatus as described in Syme et al. (2013), which was also used for the cardiac muscle/trabeculae (see below), at the fish's acclimation temperature and with the saline bubbled with pure O_2_. The stimulus voltage was adjusted for each individual muscle strip so that it produced maximal isometric force; the stimulus pulse duration ranged from 0.05 to 1 ms, and the stimulus frequency from 100 to 200 Hz. Muscle length was initially adjusted so that it produced maximum net work at 6% strain (peak-to-peak) and a cycle frequency of 2 Hz; strain was selected as being similar to values reported for red muscle measured during steady swimming in scup (Coughlin et al., 1996), and this was the contraction frequency at which the muscle produced substantial power at all temperatures. For measures of mechanical performance, preparations were subjected to a sequence of 30 cycles of working contractions, with measures taken from the last contraction in the series (i.e. where force and work had stabilized). Preparations were allowed to rest for ∼3 min between each series of measurements. Muscles were exposed to test temperatures of 2, 6, 15, 22 and 26°C in random order, and work and power were measured across a range of cycle/contraction frequencies (equivalent to tail-beat frequency) including 0.5, 1, 2, 2.5, 3, 4, 5 and 7 Hz, all at a strain of 6% peak-to-peak. For all experiments (red muscle power, spongy myocardium power and efficiency), temperature was changed over a ∼10 min interval, and preparations were left at this temperature for at least 15 min before measurements commenced. At each temperature, the specific range of cycle frequencies studied was adjusted to encompass that resulting in maximal power. However, as the skeletal muscle preparations appeared quite sensitive to exposure to 26°C, measures were only made at a cycle frequency of 3 Hz at this temperature to limit the time the muscles were exposed to this warm temperature. A value of 3 Hz was chosen as it is near where the muscle would produce maximal power at 26°C based on results at 22°C, and within 10% of maximal based on patterns at the other temperatures where the power versus frequency relationships were relatively flat near maximal power. Thus, power at 26°C was included in the analysis of the effect of temperature on maximal power, but not analyses of optimal cycle frequency. The stimulus phase (onset of stimulus relative to the imposed sinusoidal strain cycle) and the duration of the train of stimulus pulses used to activate the muscle during each cycle were adjusted at each combination of temperature and contraction rate to maximize net work output.

For experiments involving ventricular (spongy) trabeculae, the preparation of isolated bundles of muscle and the experimental apparatus and oxygenated saline were as described in Syme et al. (2013), except that preparations were tied directly to the servomotor and force transducer with 6-0 silk suture, and no pyruvate was added to the saline. Tonic adrenergic stimulation (i.e. ∼5 nmol l^−1^ epinephrine) was also omitted from these experiments given that it was not possible to maintain constant levels over the course of these lengthy and complex experiments (i.e. it would have increased data variability) (Carnevale et al., 2020). The length of the preparations was adjusted so that developed twitch force was as high as possible without a sharp rise in resting tension, and on the ascending limb near the plateau of the length–tension relationship. Preparations were subjected to working contractions with cyclic/sinusoidal strain (8% peak-to-peak) and phasic stimulation (see Syme et al., 2013 for details) over a range of contraction rates (20, 40, 60, 80, 100, 120 and 140 contractions min^−1^; equivalent to heart rate, *f*_H_) and test temperatures (2, 6, 15, 22 and 26°C). The range of contraction rates studied encompassed those that allowed for identification of maximal net power, but also always included the fastest rate at which the muscle could be activated and still follow the stimulus; this differed across temperatures and between species (see Results). Preparations underwent 30 consecutive contractions at each combination of temperature/contraction rate tested, and measures of work were taken from the last contraction in each series of 30. At each temperature/contraction rate combination, the stimulus phase and the proportion of the strain cycle period that comprised shortening (analogous to the period of systolic shortening) were adjusted to maximize net work output. This resulted in the muscle shortening during the period that it was actively contracting, and being relaxed while lengthening, similar to how the heart functions *in vivo*. For both skeletal and cardiac muscle, measures of shortening work (work done by the muscle while shortening), lengthening work (work required to lengthen the muscle) and net work (the difference between shortening and lengthening work) were converted into their respective measures of power (in W) by multiplying work done per cycle (in J) by the cycle frequency (Hz).

At the conclusion of each experiment, measurements of work were repeated at the same temperature and contraction rate as used at the start of the experiment to assess stability of the preparations. For experiments on red muscle, work at the end of the experiment averaged 100.2±4.2% of that at the start, and for experiments on ventricular myocardium work averaged 113.0±4.2%.

### Cardiac muscle efficiency

The efficiency of mechanical work was measured in ventricular cardiac muscle from 15°C acclimated fish, to test if there were differences between warm acclimated salmon versus char when exposed to different temperatures and contraction (heart) rates. Efficiency was calculated as the ratio of work done to the energy consumed by the muscle while doing work. Work was measured from muscles using the work loop technique as described above, and energy use was calculated from the oxygen consumed by the muscle, assuming that ATP production is dominated by aerobic metabolism in the fish heart (Driedzic et al., 1983). Ventricular trabecular preparations were mounted in a small, saline-filled well (244 µl volume, same saline as used for measures of mechanical performance) milled in a block of enamel coated aluminum. Water from a circulating chiller passed through tubes within the aluminum block to maintain the temperature, which was measured by a thermocouple placed into the block directly beside the well. Platinum wire pins entered the well at each end through small, polyethylene-lined holes. The ends of the muscle were tied to these pins with 6-0 silk suture, and the opposite ends of the pins, external to the well, were attached to a force transducer (model 404A, Aurora Scientific, Aurora, ON, Canada) and a servomotor (model 350, Cambridge Technology, Cambridge, MA, USA). The servomotor and force transducer were mounted to three-dimensional micro-positioning stages that allowed fine adjustment so that the pins moved freely through the holes in the ends of the well. The close fit of the pins to the polyethylene tubes minimized the diffusion of oxygen between the well and external environment, and the method of calculating oxygen consumption in working muscle (described below) accounted for any small leaks. Segments of fine, enamel-coated, magnet wire (60 µm diameter) were soldered to the platinum pins external to the well and used to stimulate the cardiac preparations. These wires were carefully draped beside the well so that they did not affect the movement of the pins. The wires were attached to a stimulator (Isostim A320, World Precision Instruments Inc., Sarasota, FL, USA) that was in turn gated by a computer. The stimulus was a 2 ms duration square voltage pulse, with the voltage adjusted to approximately 150% of that required to elicit a maximal contraction from the preparation (6–10 V). The duration of the stimulus pulse was longer than that used for measures of muscle mechanics to allow the lowest stimulus voltage possible. This avoided electrolysis on the simulating electrodes within the chamber yet still fully activated the muscles. The stimulus phase and the relative periods of the imposed sinusoidal strain pattern that comprised shortening versus lengthening were also adjusted to maximize net work output at each temperature/rate combination. Strain was 8% peak-to-peak in all cases. Muscle length was adjusted so that the muscle strips (trabeculae) produced maximal net work at 15°C and 70 contractions min^−1^.

The oxygen level in the chamber was measured with a fiber-optic oxygen probe and a 5 mm oxygen-sensitive spot (Fibox 3, PreSens; Regensburg, Germany). Once the muscle was in place in the well, the upper surface of the well was sealed with a glass microscope coverslip to which an oxygen sensor spot was affixed on the surface in contact with the saline in the well. The tip of the oxygen probe was then placed over the coverslip and sensor spot so that the oxygen partial pressure (*P*_O_2__) of the saline bathing the muscle could be measured (one recording every 10 s). The saline in the chamber was well mixed during experiments by a small, glass-coated magnetic stir bar placed at the bottom of the well, beneath the muscle preparation.

At the start of each measurement of oxygen consumption, the well was opened by removing the coverslip, fresh oxygen-saturated saline was flushed into the well, and the well was then sealed with the coverslip. The preparations were left to rest for approximately 10 min to obtain a stable baseline recording of oxygen consumption. The muscle was then stimulated and made to perform work for 200 consecutive cycles, after which the muscle was allowed to rest for 20–30 min until a stable (resting) baseline of oxygen consumption was again attained. Preliminary trials indicated that there was no difference in the rate of oxygen consumption whether 100, 200 or 300 cycles were used, suggesting that the preparations were not working anaerobically. Measures of oxygen consumption were made at temperatures of 15, 20 and 25°C, and at cycle frequencies (equivalent to *f*_H_) of 70 min^−1^ at all temperatures (*in vivo f*_H_ of Atlantic salmon and Arctic char at 15°C), 100 min^−1^ at 20°C (*in vivo f*_H_ at 20°C) and 120 min^−1^ at 25°C (*in vivo f*_H_ at 25°C) (Penney et al., 2014). This allowed for a comparison of contraction efficiency at the *in vivo f*_H_ at each temperature tested, and at a common *f*_H_ across all temperatures.

Oxygen consumed by the working muscle was calculated from the change in *P*_O_2__ of the saline above that which occurred when the muscle was resting. This was measured by fitting a linear regression to the pre- and post-working (i.e. resting) segments of the oxygen versus time relationship, and then measuring the difference in *P*_O_2__ between these two regressions at the mid-point of the series of working contractions (Syme, 1994; Trinh and Syme, 2007). This change was multiplied by the chamber volume and the oxygen solubility of the saline. The latter was obtained from standard tables (with 15.2 ppt salinity at atmospheric *P*_O_2__: 9.15 mg O_2_ l^−1^ at 15°C, 8.31 mg O_2_ l^−1^ at 20°C and 7.57 mg O_2_ l^−1^ at 25°C). The energy used by the muscle, based on the amount of oxygen used, was calculated assuming an energy equivalent of 450 kJ mol^–1^ O_2_ (Trinh and Syme, 2007).

### Data analyses

At the conclusion of all experiments, the muscle preparation was removed from the chamber, the ties and obviously dead tissue were dissected away, and the preparation was blotted on filter paper to remove surface moisture and weighed on a microbalance (Mettler UMT2, Mettler-Toledo, Columbus, OH, USA). The raw data (force and muscle length) were subject to noise filtering before analysis as described previously (Syme et al., 2013). Data for power in the graphs are expressed relative to the largest value of net power recorded for each preparation. This was done to facilitate identification of relative changes with temperature, and to avoid the inaccuracies that result from using mass-specific power caused by: (1) the unavoidable inclusion of some non-viable tissue in the dissected preparations (Syme et al., 2013); and (2) the tendency for maximal *f*_H_ to vary between some preparations. The latter would result in not all preparations being equally represented in every data point, and thus, create misleading variability and biases in the means of mass-specific power at different combinations of temperature and contraction frequency. However, mass-specific values of power are reported in Tables 3 and 4 to allow for the comparison of muscle performance between species and across acclimation temperatures.

**Table 3.**
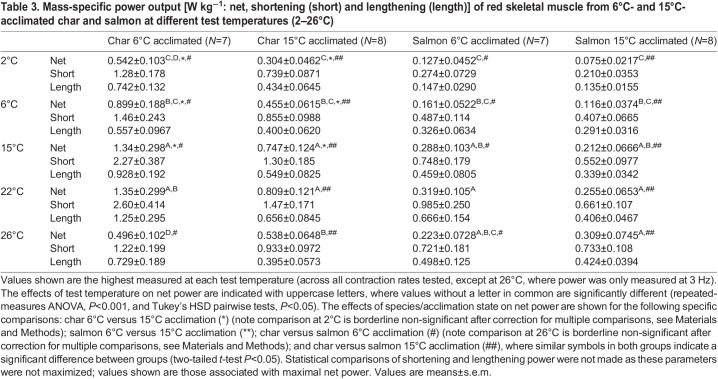
Mass-specific power output [W kg^−1^: net, shortening (short) and lengthening (length)] of red skeletal muscle from 6°C- and 15°C-acclimated char and salmon at different test temperatures (2–26°C)

**Table 4.**
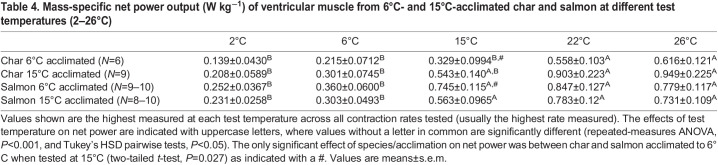
Mass-specific net power output (W kg^−1^) of ventricular muscle from 6°C- and 15°C-acclimated char and salmon at different test temperatures (2–26°C)

All data were initially assessed for equal variance and normality using Shapiro–Wilk tests. Statistical analyses included: (1) repeated-measures ANOVAs to compare the effects of temperature on power output within species/acclimation state [note, absolute values of power (W) produced by the muscle were used for these tests, not the normalized data shown in the figures]; (2) one-way ANOVAs or two-tailed *t*-tests (or Mann–Whitney rank-sum tests where the assumption of equal variance failed) to compare results between species/acclimation state at a given test temperature and contraction rate; (3) two-way ANOVAs with species and acclimation state as the main effects to compare physical characteristics of the fish and preparations that were used in the different studies (these data are shown in Tables 1 and 2); and (4) two-way ANOVAs with species and temperature as the main effects to compare the contraction frequencies at which maximal net power was produced. For comparisons between species/acclimation states (see ‘2’ above), we restricted statistical testing to only comparisons based on *a priori* hypotheses. These included testing differences between acclimation temperatures within a species (i.e. comparing char acclimated to 6°C versus 15°C, or similarly for salmon), and comparing between species at the same acclimation temperature (i.e. comparing char versus salmon when both were acclimated to either 6 or 15°C). We did not compare one species acclimated to 6°C versus the other species acclimated to 15°C as these pair-wise comparisons were not of interest or relevance.

Differences were considered significant at *P*<0.05. Where ANOVA tests indicated significant effects, subsequent comparisons were performed using Tukey's HSD multiple pairwise comparisons, which make appropriate adjustments for multiple comparisons. To account for multiple comparisons across the various ANOVAs and other independent pairwise comparisons (97 tests total), we also subjected all of these results to a Benjamini–Hochberg procedure for controlling false discovery (Benjamini and Hochberg, 1995), with the false discovery rate set at 10%. With this adjustment, two comparisons that were significant without adjustment became borderline non-significant (Table 3, adjusted critical *P*=0.046 versus actual *P*=0.05 comparing maximal net power at 26°C between char versus salmon acclimated to 6°C; and adjusted critical *P*=0.045 versus actual *P*=0.046 comparing maximal net power at 2°C between char acclimated to 6 versus 15°C). Statistical analyses were performed using SigmaPlot 12 (Systat software, San Jose, CA, USA). Values in the text, tables and graphs are given as means±s.e.m.

In the figures showing relative power, pairwise comparisons of all combinations of temperature are not shown to reduce clutter, and because the details of such individual comparisons were of less interest than the main effects themselves. However, all pairwise temperature comparisons can be found in Tables 3 and 4, where mass-specific power is reported. Tables 3 and 4 also show statistical comparisons between species and acclimation states for net power. Statistical tests of the effects of temperature on shortening and lengthening power were not performed as they did not attain maxima, making statistical comparisons of these parameters uninformative. Further, because shortening and lengthening power simply continued to increase with increased contraction rates, unlike net power, which attained maxima, values of shortening and lengthening power are provided only as Figs S1–S4.

## RESULTS

### Contractile mechanics

Physical characteristics of the fish and muscle preparations used for measures of red skeletal muscle contractile mechanics are shown in Table 1, whereas those for the ventricular muscle are shown in Table 2. Of note, while the relative ventricular mass (RVM) of the two species was not affected by acclimation temperature, the RVM of the char was approximately 35% larger than for salmon (∼0.114% versus 0.083%, *P*<0.001). Activation parameters used in the red and the ventricular muscle experiments can be found in Tables S1 and S2, respectively.

### Red muscle

**Fig. 1.**
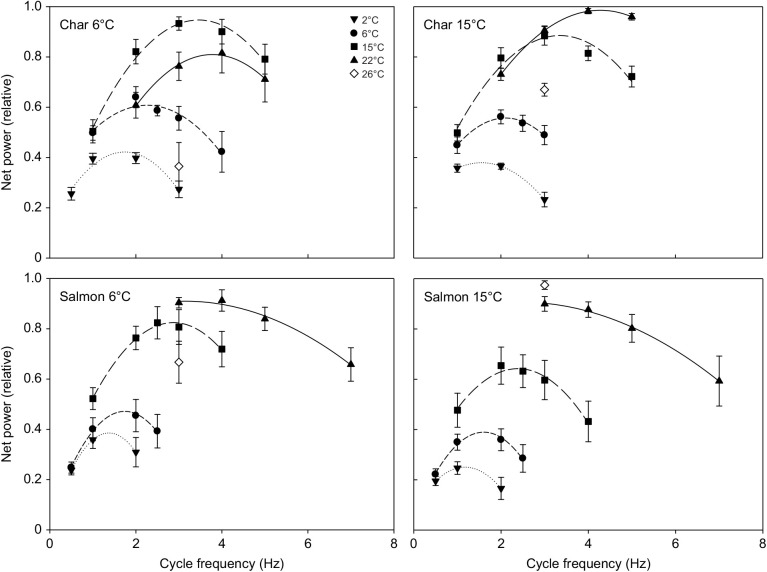
**Net power produced by the red skeletal muscle from 6°C- and 15°C-acclimated Arctic char and Atlantic salmon at a range of test temperatures and contraction frequencies.** Power is expressed relative to the maximal net power produced for each preparation. The key shows the test temperature at which measurements were made. Each panel shows results for a given species and acclimation state (char 6°C acclimated *N*=7; char 15°C acclimated *N*=8; salmon 6°C acclimated *N*=7; salmon 15°C acclimated *N*=9). Values are means±s.e.m. See Table 3 for mass-specific values and statistical comparisons between temperatures. There was a significant effect of temperature on maximal net power (i.e. the peak values attained, *P*<0.001) and on the contraction frequency at which maximal power was attained (*P*<0.001) in all species and acclimation states.

**Fig. 2.**
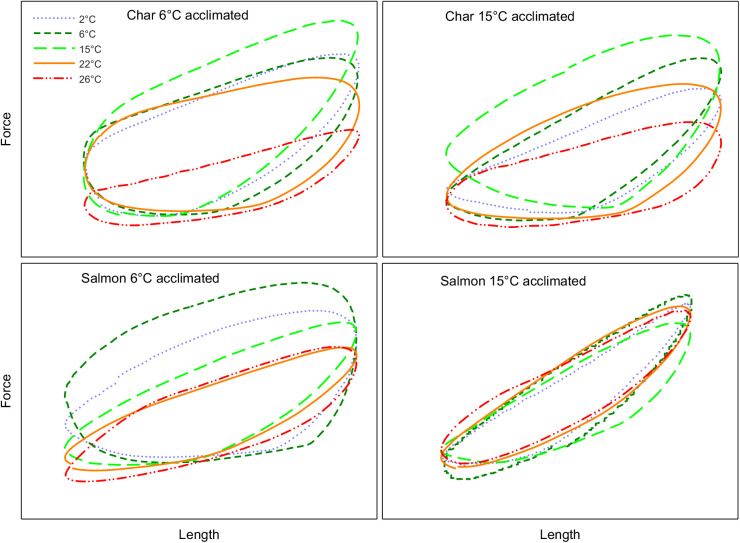
**Representative work loops from the red skeletal muscle of cold- and warm-acclimated Arctic char and Atlantic salmon at different temperatures.** The work loops shown at each temperature are at the contraction rate that resulted in maximal net power output. The contraction rates are as follows, in the sequence 2/6/15/22/26°C: 6°C-acclimated char 2/2/3/4/3 Hz; 15°C-acclimated char 1/1/2/3/3 Hz; 6°C-acclimated salmon 1/1/3/4/3 Hz; 15°C-acclimated salmon 1/1/2/3/3 Hz.

Net power rose to a maximum (maximal power) and then declined with increasing cycle frequency in all groups (Fig. 1). The cycle frequency at which net power was maximal (optimal frequency) was greater at higher test temperatures (*P*<0.001 in both species and at both acclimation states), and was also higher in char than in salmon at either acclimation temperature (two-way ANOVA with temperature and species as main effects, *P*<0.001 at both acclimation temperatures). Maximal power was also temperature dependent (*P*<0.001 in both species and both acclimation states). However, maximal power was not always monotonically dependent on test temperature, with the pattern of response being related to the acclimation state and species (Fig. 1, Table 3). In cold-acclimated char, maximal net power increased with temperature up to 15°C (*P*<0.05), but then declined substantially such that power at 26°C was not different from maximal power at 2°C (*P*=0.74). In warm-acclimated char, maximal power increased with temperature up to 22°C, and declined less substantially such that power at 26°C was greater than at 2°C (*P*=0.003) but not at 6°C (*P*=0.61). However, despite this apparent improvement in relative performance at warmer test temperatures with warm acclimation, maximal mass-specific net power from muscle of warm-acclimated char was 40–50% lower than that from cold-acclimated char between 2 and 15°C (Table 3). At 22 and 26°C there was no impairment, but also no improvement, resulting from warm acclimation. Net power from salmon red muscle showed similar patterns of response to those for char, but differed in some important ways (Fig. 1, Table 3). First, mass-specific net power in the char was 2- to 5-fold higher than in similarly acclimated salmon at most test temperatures. Second, power in cold-acclimated salmon continued to increase up to 22°C (versus 15°C in char) before falling at 26°C, and fell less substantially than in char. Third, this greater tolerance for warm temperatures was even more evident in warm-acclimated salmon, where power was maintained up to 26°C. Fourth, mass-specific power was not significantly lower in warm- versus cold-acclimated salmon at any test temperature. Many of these effects are evident in the presented work loops (Fig. 2), where changes in net work (i.e. the area of the loop) with temperature were associated with changes in the ability of the muscle to produce and sustain force (being mindful that power is a function of both work and contraction rate). Red muscle from cold-acclimated char exhibited a very large drop in work-loop area at 26°C, but much less of a drop when warm acclimated. In cold-acclimated salmon, work-loop area changed relatively little between 15 and 26°C, and not at all in warm-acclimated salmon. In combination with increased contraction rates with increased temperature, these changes in work-loop area translated into a substantial drop in power at warm temperatures in char, and more so in cold-acclimated fish, but little or no decrement in salmon, particularly warm-acclimated fish.

The relative shortening and lengthening power of red muscle from both species, and at both acclimation temperatures, increased monotonically and near-linearly with increasing cycle frequency over the range studied (i.e. a range that resulted in net power attaining a maximum in most cases), by 5-fold for shortening power and by 10-fold for lengthening power in both species (Table 3, Figs S1 and S2). Shortening and lengthening power at any given contraction rate were also quite insensitive to test temperature in both char and salmon. On average: (1) mass-specific shortening power from salmon muscle was less than half that of char; and (2) mass-specific shortening power in warm-acclimated char was reduced compared with cold-acclimated char at all but the warmest test temperatures, whereas salmon muscle contractile function was not impaired by warm acclimation (Table 3).

### Ventricular muscle

**Fig. 3.**
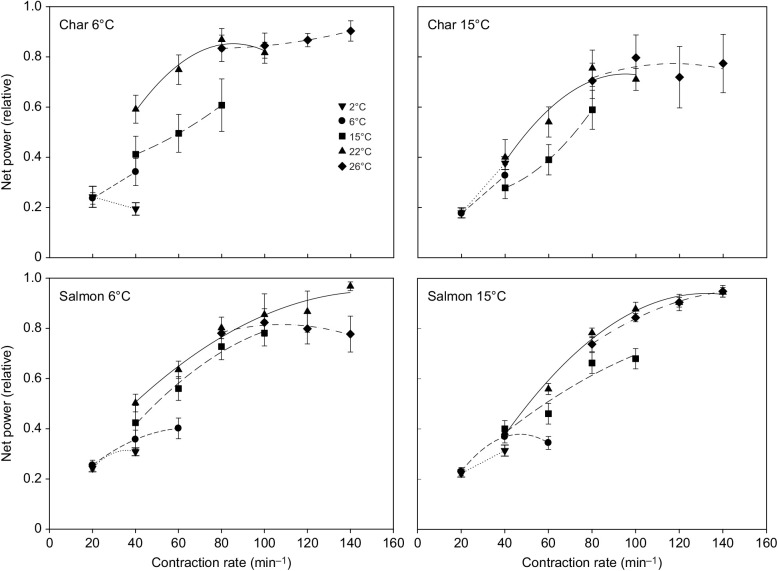
**Net power produced by ventricular muscle strips (trabeculae) from 6°C- and 15°C-acclimated Arctic char and Atlantic salmon at a range of test temperatures and contraction frequencies.** Power is expressed relative to the maximal net power produced for each preparation. The key shows the test temperature at which measurements were made. Each panel shows results for a given species and acclimation state (char 6°C acclimated *N*=8, char 15°C acclimated *N*=9, salmon 6°C acclimated *N*=10, salmon 15°C acclimated *N*=11). The highest contraction rate shown at each test temperature is the fastest the heart could be paced and still follow the stimulus, and is thus the maximal heart rate achievable at that temperature. Values are means±s.e.m. For mass-specific values and statistical comparisons, see Table 4. There was a significant effect of temperature on maximal net power (i.e. the largest values attained, *P*<0.001) and on the contraction frequency at which maximal power was attained (*P*<0.001) in all species and acclimation states.

**Fig. 4.**
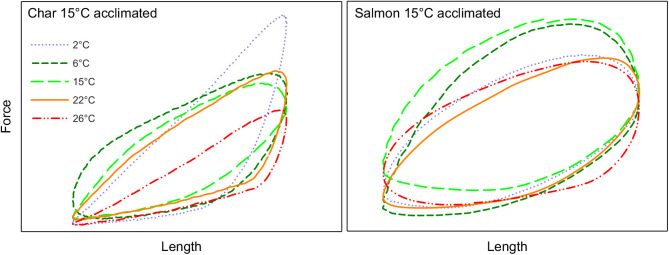
**Representative work loops from the ventricular myocardium of 15°C-acclimated Arctic char and Atlantic salmon at different temperatures.** The work loops shown at each temperature are at the contraction rate that resulted in maximal net power output. The contraction rates (min^−1^), in the sequence 2/6/15/22/26°C, are as follows: char 20/40/80/80/120 min^−1^; salmon 40/40/80/140/140 min^−1^. Note: work loops in 6°C- and 15°C-acclimated fish were similar.

There were a number of differences between how ventricular muscle responded to increases in test and acclimation temperature compared with the red skeletal muscle. (1) In all groups, net power tended to increase with contraction rate up to the highest rates that the myocardium could sustain (Fig. 3, Table 4). (2) In all groups, maximal relative (Fig. 3) and mass-specific (Table 4) net power first increased, but then attained a maximum and did not then fall as test temperature was increased from 2 to 26°C (*P*<0.001). The increase across this temperature range was approximately 4.5-fold in char versus 3.5-fold in salmon. (3) The warmer acclimation temperature did not affect/impair myocardial net power in either species. (4) There was only one instance where mass-specific myocardial power of char differed from that of salmon (fish acclimated to 6°C when tested at 15°C) (Table 4). These effects of temperature are evident in the work loops presented in Fig. 4 (note: only loops from 15°C acclimated fish are shown as there was not an acclimation effect on myocardial power). The area of the loops begins to fall at the higher temperatures, but this is associated with increased rates of contraction so that maximal net power does not fall or continues to rise with increased temperature in both species.

Similar to net power, the shortening and lengthening power of ventricular muscle increased with contraction rate, and was maintained even at the warmest temperature tested, in both warm- and cold-acclimated char and salmon (Figs S3 and S4, Table 4). However, the relative increase in lengthening power with increased contraction rate in warm-acclimated char was much greater (approximately double) than in cold-acclimated char, suggesting a stiffening of ventricular muscle in warm-acclimated fish. Overall, test temperature appeared to have only a small effect on shortening power at any contraction rate.

Ventricular muscle from salmon was able to sustain higher contraction rates than muscle from char at most temperatures (Fig. 3, the highest rates shown are the highest rates the muscles could sustain). For example, in warm-acclimated fish: salmon trabeculae at 6°C could contract up to 60 min^−1^ whereas char could only contract up to 40 min^−1^; at 15°C, salmon trabeculae could contract up to 100 min^−1^ but char only to 80 min^−1^; at 22°C, none of the char preparations could follow the stimulus beyond 100 min^−1^ while all salmon preparations could contract up to 140 min^−1^; and at 26°C, only five of nine char preparations could follow 140 min^−1^ stimulus whereas all nine of the salmon preparations were capable of contraction at this rate.

### Contractile efficiency

The physical characteristics of the fish and ventricular trabecular preparations, and the stimulus phases and the strain cycles used for each test condition, are shown in Table 2 and Table S3, respectively. The *P*_O_2__ of the saline in the chamber at the start of each measurement was consistently near 65 kPa (Table 5), and not significantly different between any of the groups (*P*=0.239). This is approximately 3-fold greater than atmospheric *P*_O_2__, but was set at this level to eliminate potential diffusion limitations within the muscle preparations, and to ensure adequate oxygen remained in the saline over the course of recordings. Resting metabolic rate was similar between species and test temperatures (*P*=0.152), and was very similar before and after the bout of work was completed; i.e. the resting metabolic rate before measurements of work was within 5% (95–101%) of that at the end (Table 5).

**Table 5.**
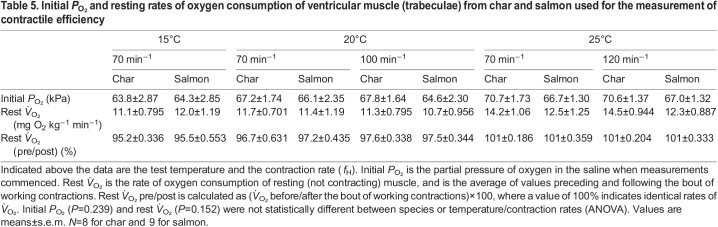
Initial *P*_O_2__ and resting rates of oxygen consumption of ventricular muscle (trabeculae) from char and salmon used for the measurement of contractile efficiency

**Fig. 5.**
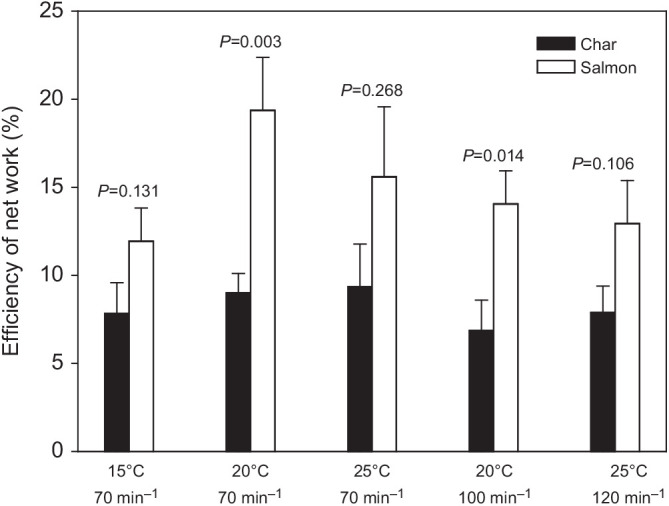
**Efficiency of net work produced by the spongy ventricular myocardium of Arctic char and Atlantic salmon acclimated to 15°C.** Efficiency was measured at 15°C and 70 min^−1^, 20°C and 100 min^−1^, and 25°C and 120 min^−1^, which are the heart rates associated with these body temperatures *in vivo*, and at a fixed heart rate of 70 min^−1^ at 15, 20 and 25°C to allow comparison of the effects of temperature alone (char *N*=8, salmon *N*=9). *P*-values above the bars show comparisons between char and salmon at each temperate/heart rate combination. Temperatures did not affect efficiency in either species when assessed at the *in vivo* heart rate (*P*=0.589 in salmon, *P*=0.765 in char, repeated-measures ANOVA). Temperature had a significant effect on efficiency measured at 70 min^−1^ in salmon (*P*=0.030, repeated-measures ANOVA; 20°C greater than 15°C, *P*<0.05, Tukey’s test), but not in char (*P*=0.612, repeated-measures ANOVA). Values are means±s.e.m.

The efficiency of net work was expressed as the ratio of net work done (i.e. the net mechanical work done by the muscle during a complete cycle of lengthening and shortening, equivalent to filling and ejection in a beating heart) relative to the metabolic cost of doing that work above resting values (both measured in J). It is thus a measure of the relative cost of doing work over a complete cardiac cycle. Efficiency of net work (Fig. 5) was not affected by contraction rate (*f*_H_) in either salmon or char (*P*=0.589 RM ANOVA for salmon comparing 70 versus 100 versus 120 min^−1^ at the *in vivo* temperatures associated with these heart rates, 15, 20 and 25°C, respectively; *P*=0.765 RM ANOVA for char with similar parameters as for salmon). However, with *f*_H_ fixed at 70 min^−1^, temperature had a significant effect on ventricular efficiency in salmon (*P*=0.030 RM ANOVA), with efficiency at 20°C being greater than at 15°C (*P*<0.05 Tukey's HSD test), but no effect in char (*P*=0.612 RM ANOVA) (Fig. 5). Further, the efficiency of work in salmon ventricular muscle was consistently higher than in char, by approximately 2-fold, when tested at 20°C at either 70 or 100 min^−1^ (*P*=0.003 and 0.014, respectively) (Fig. 5).

## DISCUSSION

### Red muscle contractile performance

In the red muscle, the cycle frequency at which net power was maximal (i.e. the optimal cycle frequency) increased with temperature (Fig. 1 and Results), similar to what has been observed in other studies on fish red muscle (e.g. Johnson and Johnston, 1991; Rome and Swank, 1992; Altringham and Block, 1997). Likewise, tail beat frequency in swimming fish typically increases with ambient water temperature (e.g. Nudds et al., 2020 and references therein). This increase in optimal frequency and/or tail beat frequency might allow fish to benefit from increased muscle power. However, whether it does is not entirely clear. Although an increase in tail beat frequency tends to maintain the Strouhal number in a range for economical thrust production, it is also often associated with a decrease in tail-beat amplitude that would constrain power (Nudds et al., 2020).

Muscle power also increased with temperature across much of the range studied, although at higher temperatures net power attained a plateau or began to decline (Fig. 1, Table 3). This is similar to what might be expected based on a rise, and then fall, in sustained swim speeds with increased temperature (Fry and Hart, 1948). Warm acclimation conferred some improved ability to operate at warm test temperatures in both species (Fig. 1, Table 3). Yet the relatively eurythermal salmon appears better suited than the more stenothermal char to sustain or increase muscle power for swimming at warmer temperatures when acclimated to either temperature, and the salmon benefits from warming even to 26°C when acclimated to 15°C. In fact, our data suggest that char red muscle function/swimming performance would be impaired at temperatures above 15°C when cold acclimated, and above 22°C even when warm acclimated. Similarly, Shuman and Coughlin (2018) noted that the acclimation responses of muscle contractile capacity were greater in the eurythermal rainbow smelt (*Osmerus mordax*) compared with the more stenothermal rainbow trout, to the extent that there appeared to be a lack of acclimation response in red skeletal muscle function in the trout (Coughlin et al., 2020).

Changes in muscle myosin isoform expression and contraction speed following cold acclimation in fish typically result in improved performance at these temperatures (Johnston and Temple, 2002), and similarly, our results suggest that warm acclimation generally improves or maintains contractile performance at high temperatures (Table 3). However, warm acclimation is not always associated with improved performance. For example, the maximal isotonic power of carp (*Cyprinus carpio*) red muscle is not improved, only maintained, in warm-acclimated fish when operating at warm temperatures, and isometric force is reduced (Johnston et al., 1985). Warm acclimation does not impair, but also does not improve, burst or sustained swimming performance in mosquito fish (*Gambusia holbrooki*) (Wilson et al., 2007). Finally, Coughlin et al. (2020) noted no changes in red muscle myosin isoform expression in rainbow trout red muscle when acclimated to 10 versus 20°C, and little to no changes in red muscle contractile capacity in warm-acclimated fish.

Although the above patterns of changes in power output with temperature suggest some improvement in the tolerance of red muscle to high temperatures following warm acclimation, the mass-specific power output of muscle from warm-acclimated char was reduced by ∼50% compared with cold-acclimated fish at the cooler test temperatures (2–15°C), and such an effect was not seen in salmon (Table 3). The factor(s) mediating this decrease in mass-specific power at cooler temperatures following warm acclimation in char are unclear. Although the warm acclimation temperature (15°C) was well below the CT_max_ of either species, this temperature may still be stressful to char as they prefer cooler temperatures in the range of 0–16°C, whereas salmon are found at temperatures ranging from 0 to 28°C (Caissie et al., 2014; Strøm et al., 2020). This temperature-dependent response could have resulted from changes in the contractile proteins (Watabe, 2002) and in muscle fibre types (Hammill et al., 2004). For example, myofibrillar ATPase activity is reduced by warm acclimation in goldfish, and only converges on that in cold-acclimated fish at warmer temperatures (Johnston et al., 1975). However, it is unlikely to be related to remodeling of the tissue that affected contractile protein density (as is associated with force and power in high-speed synchronous muscles; Syme and Josephson, 2002), because warm-acclimated char muscle was still able to maintain high power at the warmer test temperatures. Perhaps the contractile performance of the muscle was constrained, such that the reduced power following warm acclimation is an energy sparing mechanism. Overall, our results show that warm acclimation does not convey the same benefits to operating at warm temperatures as cold acclimation does to functioning in the cold.

It was also observed that the red muscle of salmon only produced about one-quarter of the mass-specific power of muscle from char at both acclimation states (Table 3). It is possible, but very unlikely, that this reflects systematic differences in assessing the mass of viable muscle tissue in the preparations between the two species. The difference is more likely to have some basis in char red muscle having higher optimal frequencies for net power than salmon (Fig. 1 and Results), where higher contraction rates in char would contribute to increased capacity to produce power. If the differences are real, and char skeletal muscle is indeed capable of producing substantially more mass-specific power than salmon, this would have significant implications for swimming performance, including an ability to cope with very cold water in char (i.e. enhanced mass-specific capacity to generate power in char may help compensate for cold-induced depression of muscle power). This would be functionally similar to fish recruiting more or faster fibre types in cold temperatures to sustain power output (reviewed by Syme, 2006).

### Ventricular muscle contractile performance

The net and shortening power produced by ventricular muscle tended to rise with contraction rate (*f*_H_), up to the highest stimulus rates that the myocardium could sustain (Fig. 3, Fig. S3). This pattern of increased power with *f*_H_ is similar to that observed in other studies using isolated ventricular muscle from ectotherms (Syme, 1993; Harwood et al., 2002; Syme et al., 2013), although a clear fall in power at high contraction frequencies has been observed in the rainbow trout ventricular myocardium (Shiels et al., 1998). This disparity might reflect different abilities to follow high contraction frequencies between fish species. However, it is more likely related to the approach used in the present study of adjusting the temporal pattern of applied strain at different contraction frequencies so that the duration of muscle contraction matched the duration of muscle shortening to better reflect how a beating heart functions. The observation that char myocardium became arrhythmic and failed to respond at higher stimulation frequencies, even at warm temperatures (Fig. 3 and Results), is consistent with data from a Greenland population of Arctic char acclimated to 7°C, where maximum *f*_H_ (62 beats min^−1^) was attained at 13°C and hearts became arrhythmic at 15°C on average (Hansen et al., 2017). Further, Gilbert et al. (2020) showed that the maximum heart rate of anadromous Arctic char from Nunavut (Canada) began to plateau above ∼16°C, reached a maximum at ∼19°C, then declined and became arrhythmic at ∼21°C. While ventricular muscle from salmon also became arrhythmic at high stimulation rates, it was able to attain slightly higher maximal contraction rates than char at most temperatures, in both acclimation states (Fig. 3). This may provide salmon hearts with more scope to increase cardiac power output than char, although the increase in power attained by salmon ventricular muscle when operating at these higher rates, relative to the maximal rates that could be attained by char, was only approximately 10%.

With increased temperature, the ventricular myocardium could operate at higher contraction rates, and the maximal net power attained by the myocardium tended to increase with temperature across the full range studied (*P*<0.001 for both species/acclimation states) (Fig. 3), despite an eventual decline in the net work done per cycle at higher temperatures and contraction rates (Fig. 4). Further, in contrast to previous results for the ventricular muscle of rainbow trout (Shiels et al., 1998) and cod (Syme et al., 2013), where power at a given contraction rate tended to be less at higher temperatures, shortening power from the ventricular muscle tended to be largely independent of temperature at a given contraction rate (Fig. S3). Again, this latter difference likely reflects the approach used in the present study, where the strain pattern was adjusted at each temperature and frequency so that the period of muscle shortening always closely matched the period over which contractile force was generated to better mimic what occurs in a beating heart. With this refinement in technique, power output of ventricular muscle at a given contraction frequency (*f*_H_) is not highly sensitive to temperature, and maximal power tends to increase with temperature. This limited temperature sensitivity of power output from the myocardium is likely associated with the relatively slow contraction rates of cardiac muscle in most fish, similar to the low thermal sensitivity of power in red muscle of very large fishes with relatively slow tail beat frequencies (Stoehr et al., 2020). Likewise, the lengthening power of ventricular muscle was not very temperature dependent (Fig. S4). However, acclimation to 15°C resulted in a substantial increase in relative lengthening power in char, but not salmon. Thus, ventricular filling, which is constrained by the energy required to lengthen ventricular muscle during diastole, may be compromised in warm-acclimated char.

In contrast to the reduction in mass-specific power from red skeletal muscle in warm-acclimated char, warm acclimation did not affect mass-specific net power of the ventricular muscle from char or salmon at any test temperature (Table 4). These data strongly suggest that warm acclimation in both species was not simply a stressor causing muscle impairment, and that the contractile capacity of the red skeletal muscle in char may have been actively downregulated (perhaps to reduce energy demands), while that of cardiac muscle was defended to accommodate the unavoidable increase in metabolic rate associated with warm temperatures. Further investigation into the physiological basis of the changes in muscle performance following warm acclimation is needed to address these questions.

### Efficiency of ventricular muscle

#### Effects of contraction rate

In mammals, it is well established that enthalpy (heat+work) per contraction is independent of contraction rate, and thus that energy use tends to increase in direct proportion to contraction rate (e.g. Laurent et al., 1956; Berglund et al., 1958). However, the mechanical work done per contraction can vary with contraction rate in certain preparations and/or species, and seemingly be independent of oxygen use. For example, Baxi et al. (2000) and Mellors and Barclay (2001) report that the efficiency of rat papillary muscles can be dependent on contraction rate, and Harwood et al. (2002) report a rise, and then fall, in net efficiency of rainbow trout ventricular muscle with increased contraction frequency (efficiency ranging between 15 and 25% at 15°C). In the present study, the efficiency of ventricular muscle from salmon and char was approximately 15% and 7%, respectively, and independent of contraction rate (*P*=0.95 comparing different contraction rates for char, and *P*=0.43 for salmon; Fig. 5). The independence of efficiency from contraction rate is consistent with Syme (1994), who measured net efficiencies of about 11% at 20°C across a range of contraction frequencies in the ventricular muscle of leopard frogs (*Rana pipiens*), and with studies that used intact hearts of teleost fishes. These studies showed that cardiac power and rates of oxygen consumption tend to change in concert with *f*_H_, so that efficiency is generally independent of *f*_H_ or tracks changes in work done per contraction, the latter being associated with changes in preload or afterload on the heart (Farrell, 1984; Farrell et al., 1985; Houlihan et al., 1988; Graham and Farrell, 1990; Davie and Franklin, 1992). It thus remains somewhat equivocal how, and whether, contraction rate impacts the efficiency of the working fish myocardium. Yet, even if the relative cost of pumping blood remains constant, increased *f*_H_ generally results in increased rates of power and thus ventricular oxygen consumption, and this would be of consequence to the metabolic budget of the fish and the ability of the myocardium to obtain enough oxygen to fuel the heart's contraction.

#### Effects of temperature

Temperature did not affect the efficiency of myocardial work in char, but in salmon, efficiency at 20°C was higher as compared with 15°C (Fig. 5). Thus, short-term temperature changes over the approximately 10°C range that char and salmon might expect to encounter in nature (Gilbert et al., 2016; Corey et al., 2017) could affect the efficiency of salmon hearts and perhaps influence their thermal niche. The increased efficiency of the salmon myocardium at 20°C does not appear to have a basis in the effects of temperature on mitochondrial leak or respiratory control ratio (RCR), as Penney et al. (2014) observed no effect on these parameters over a similar range of temperatures in char or salmon, and Gerber et al. (2020, 2021) found no difference in Complex I- and II-fueled RCR values in mitochondria from salmon acclimated to 12°C and assayed at 20°C. We are not aware of other reports on the effects of temperature on the efficiency of isolated cardiac muscle in fish, but there are reports from working fish hearts acclimated to different temperatures. Graham and Farrell (1990) reported that working rainbow trout hearts from fish acclimated to and working at 5°C had a reduced efficiency relative to fish acclimated to and working at 15°C (when working at the same mass-specific power output at both temperatures), although there was considerable overlap between the groups and the statistical significance of the difference is uncertain. Graham and Farrell (1990) suggest that this difference is related to reduced work per contraction and cardiac hypertrophy in cold-acclimated fish. Conversely, Farrell et al. (1985) observed a small increase in the efficiency of working hearts of winter versus summer sea raven (*Hemitripterus americanus*), perhaps related to higher *f*_H_ values and increased rates of oxygen consumption in summer fish. Thus, the effects of temperature on the contractile efficiency of fish ventricular myocardium may be species dependent, and studies on working fish hearts suggest that other factors associated with temperature may be important in impacting the costs, work and, thus, efficiency of the heart. Nonetheless, there is a dearth of information about these relationships.

Efficiency was notably lower in the char myocardium versus that of the salmon at all combinations of temperature and *f*_H_, although the difference only reached statistical significance when the preparations were working at 20°C (Fig. 5). A greater amount of energy required by ventricular muscle to perform a given amount of work in char might reflect reduced efficiency of the transduction of chemical energy into work by the contractile apparatus. However, in the present study, net work was maximized in each preparation, and based on pressure–volume area and oxygen consumption relationships in working hearts, this should have maximized contractile efficiency (Toorop et al., 1988; Westerhof, 2000). The lower efficiency in char might also reflect reduced mitochondrial coupling (Iftikar and Hickey, 2013; Rodnick et al., 2014; Gerber et al., 2020). In support of the latter hypothesis, Penney et al. (2014) showed that the State 2 and 4 respiration of cardiac mitochondria were consistently higher in 10°C-acclimated char than Atlantic salmon when measured at temperatures from 20 to 28°C, and that this resulted in 35% lower values for RCR between 20 and 24°C in char. Although myocardial contractile efficiency in char did not appear to be affected or impaired by warmer temperatures, the much lower efficiency in char versus salmon would limit their ability to meet the metabolic demands associated with high temperatures, and contribute to their reduced upper temperature tolerance (CT_max_; Penney et al., 2014).

### Conclusions and perspectives

Our findings have important implications for the ability of char and salmon to tolerate warming environments. The effects of acclimation and test temperature suggest that char inhabiting cooler waters (e.g. 6°C) would experience a substantial impairment in the capacity of red skeletal muscle to produce power, and thus swimming ability, if they were acutely exposed to temperatures of ∼15°C. Although acclimation to 15°C appears to lessen this impairment, temperatures above 22°C would still diminish muscle power in this species. Salmon in either acclimation state, however, would experience either increased power or little-to-no impairment when exposed to temperatures up to at least 26°C. Further, warm acclimation in char, but not in salmon, resulted in reduced mass-specific power output of the red muscle. Thus, long-term exposure to warmer environments could actually constrain, rather than enhance, the char's tolerance to heat. This is a potential concern with regards to climate change, and suggests that this species may have limited phenotypic plasticity in this regard. Alternatively, the downregulation of muscle power in warm-acclimated char may be a strategy to conserve energy when chronically exposed to a warm, and thus energetically stressful, environment. Regardless, the Atlantic salmon appears better suited than the Arctic char to inhabit warm environments in the context of maintaining muscle power for swimming, both acutely and chronically. In contrast, the much higher mass-specific power of char versus salmon red muscle suggests that the former species has a greater swimming capacity. This interspecific difference could provide the char with an improved capacity to swim in cold waters where the power of muscle is typically reduced and/or constrained. Other factors such as the temperature at which the muscle operates and the amount of red muscle actually recruited while swimming and available in each species need to be considered/determined before the relevance of this data can be fully appreciated.

Regarding the responses of the ventricular myocardium, the relative insensitivity of shortening power to temperature when measured at a given contraction rate (i.e. heart rate), and the continual increase in maximal myocardial power output with increasing test temperatures up to 22°C, would support elevated cardiac output in the face of increased temperatures and hence metabolic demands. This was apparent in both species and acclimation states, across a wide range of temperatures, and perhaps represents an essential response for surviving occasional, transient warming events (i.e. diurnal changes in summer stream temperatures or heat waves). Nonetheless, it is very likely that: (1) the inefficiency of myocardial respiration in char (present study; Penney et al., 2014); (2) the ability of salmon, but not char, to enhance myocardial contraction efficiency at 20 versus 15°C; (3) the increased work required to lengthen the char myocardium following warm acclimation; and (4) the constrained upper myocardial contraction frequency (consistent with Penney et al., 2014: salmon ∼134 beats min^−1^; char ∼116 beats min^−1^) would limit cardiac performance at higher temperatures in char. This conclusion is consistent with numerous studies showing that cardiac function is a key determinant of the maximum temperatures at which fish can survive (Wang and Overgaard, 2007; Farrell et al., 2009; Eliason et al., 2011; Franklin et al., 2013; Iftikar and Hickey, 2013; Eliason and Anttila, 2017; Christen et al., 2018; Leeuwis et al., 2021). Whether the difference in maximum heart (contraction) rate between char and salmon is related to interspecific differences in pacemaker properties, or to how temperature affects the ability of char ventricular myocytes to maintain ion fluxes and myocardial excitability, would be interesting to examine given the findings of Haverinen and Vornanen (2020).

While the relatively bigger hearts of char (Table 2), consistent with Anttila et al. (2015), and the greater power produced by the red skeletal muscle of this species may be important adaptations to cold temperatures, these adaptations do not appear to translate into performance at high temperatures, even when this species is acclimated to 15°C. This is consistent with the significantly lower upper thermal tolerance of adult char (CT_max_ 21–23°C) versus salmon (CT_max_ ∼26°C) (Penney et al., 2014; Gilbert et al., 2016, 2020; Christen et al., 2018; Leeuwis et al., 2019). Given that anadromous Arctic char are currently exposed to summer temperatures as high as 20°C (Gilbert et al., 2016), that a recent study by Layton et al. (2021) reported that Arctic char populations in southern Labrador have a reduced adaptive capacity (evolutionary potential), and that Atlantic salmon (which perform much better at high temperatures) are moving northward on Canada's east coast (Reist et al., 2006 and references herein), it appears that char will have no choice but to move to higher latitudes without significant conservation efforts. These efforts will need to focus on strategies and methodologies to limit further increases in water temperatures in key fluvial systems, and to prevent Atlantic salmon from establishing themselves in ecosystems and freshwater systems where they may outcompete char.

## Supplementary Material

Supplementary information
