# Supplementary material for: Temperature effects on the contractile performance and efficiency of oxidative muscle from a eurythermal versus a stenothermal salmonid
Source: J Exp Biol. 2021 Aug 5;224(15):jeb242487. doi: 10.1242/jeb.242487 (PMC8353165; doi:10.1242/jeb.242487)
Supplement: Supplementary information [file jexbio-224-242487-s1.pdf]

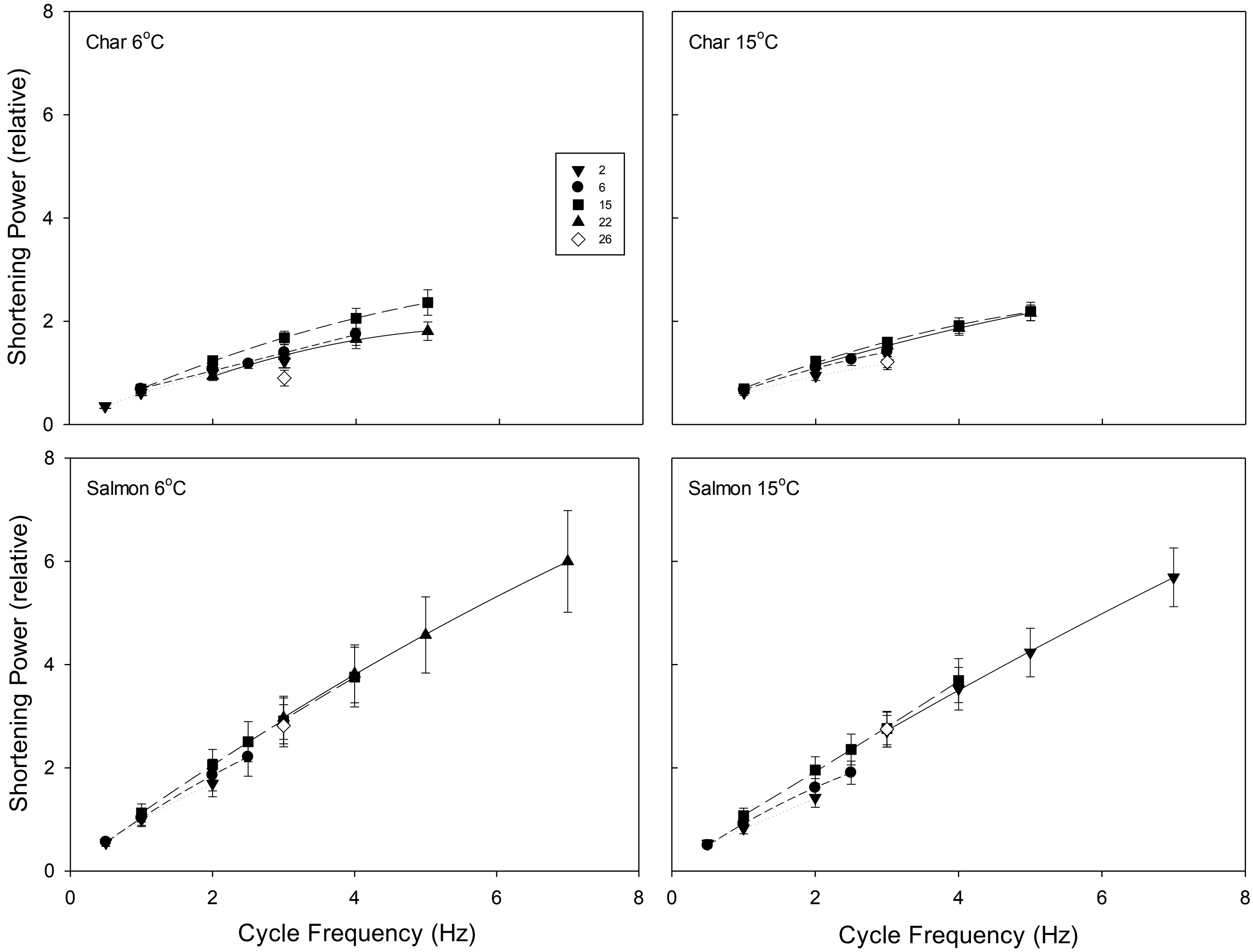

Fig. S1. Shortening power produced by the red skeletal muscle from 6oC and 15oC acclimated Arctic char and Atlantic salmon at a range of test temperatures and contraction frequencies. Statistical tests were not performed because shortening power did not attain a maxima. See Fig. 1 for additional details.

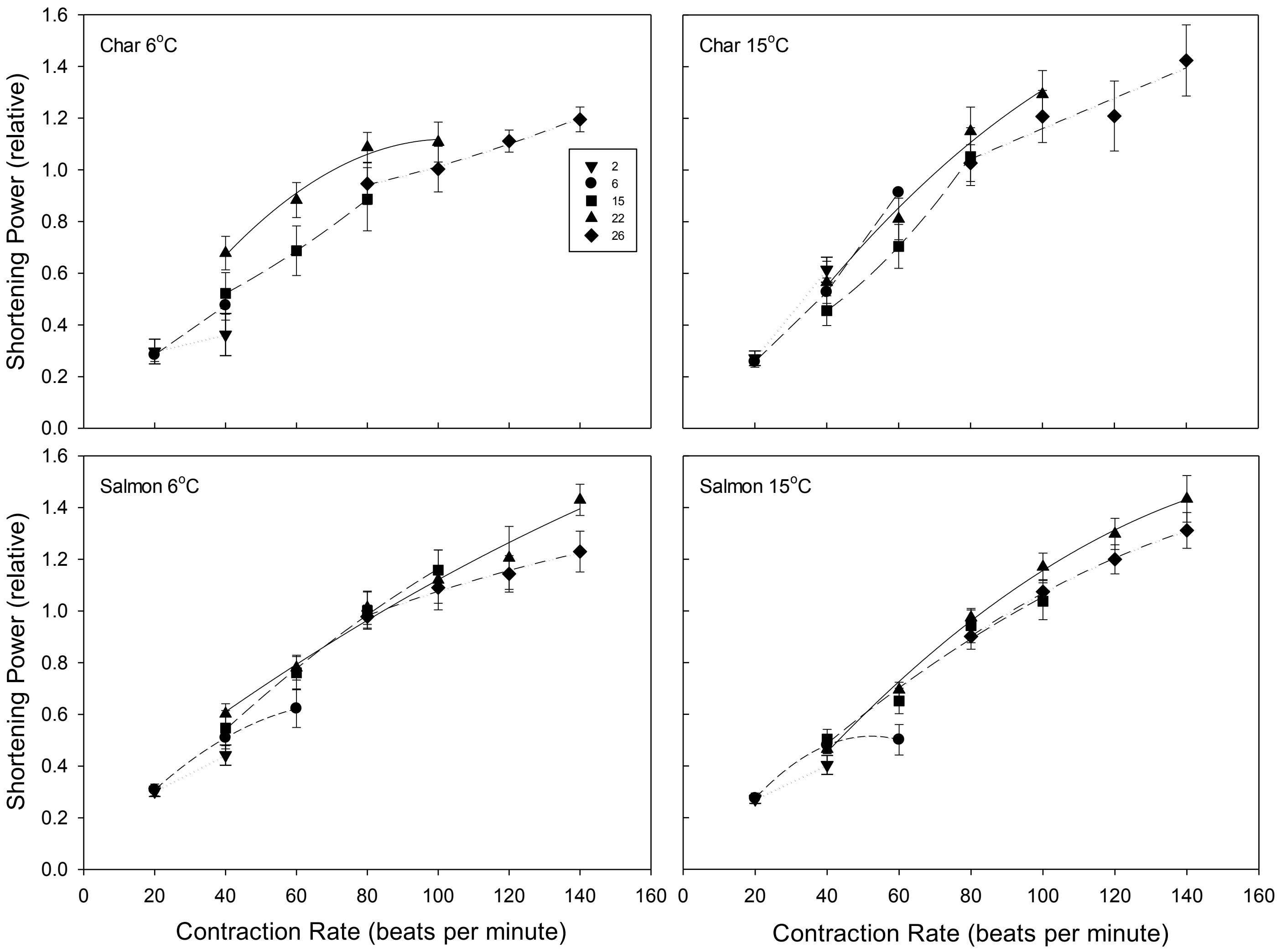

Fig. S2. Lengthening power (i.e., that required to stretch the muscle) of red skeletal muscle from 6oC and 15oC acclimated Arctic char and Atlantic salmon at a range of test temperatures and contraction frequencies. Statistical tests were not performed because lengthening power did not attain a maxima. See Fig. 1 for additional details.

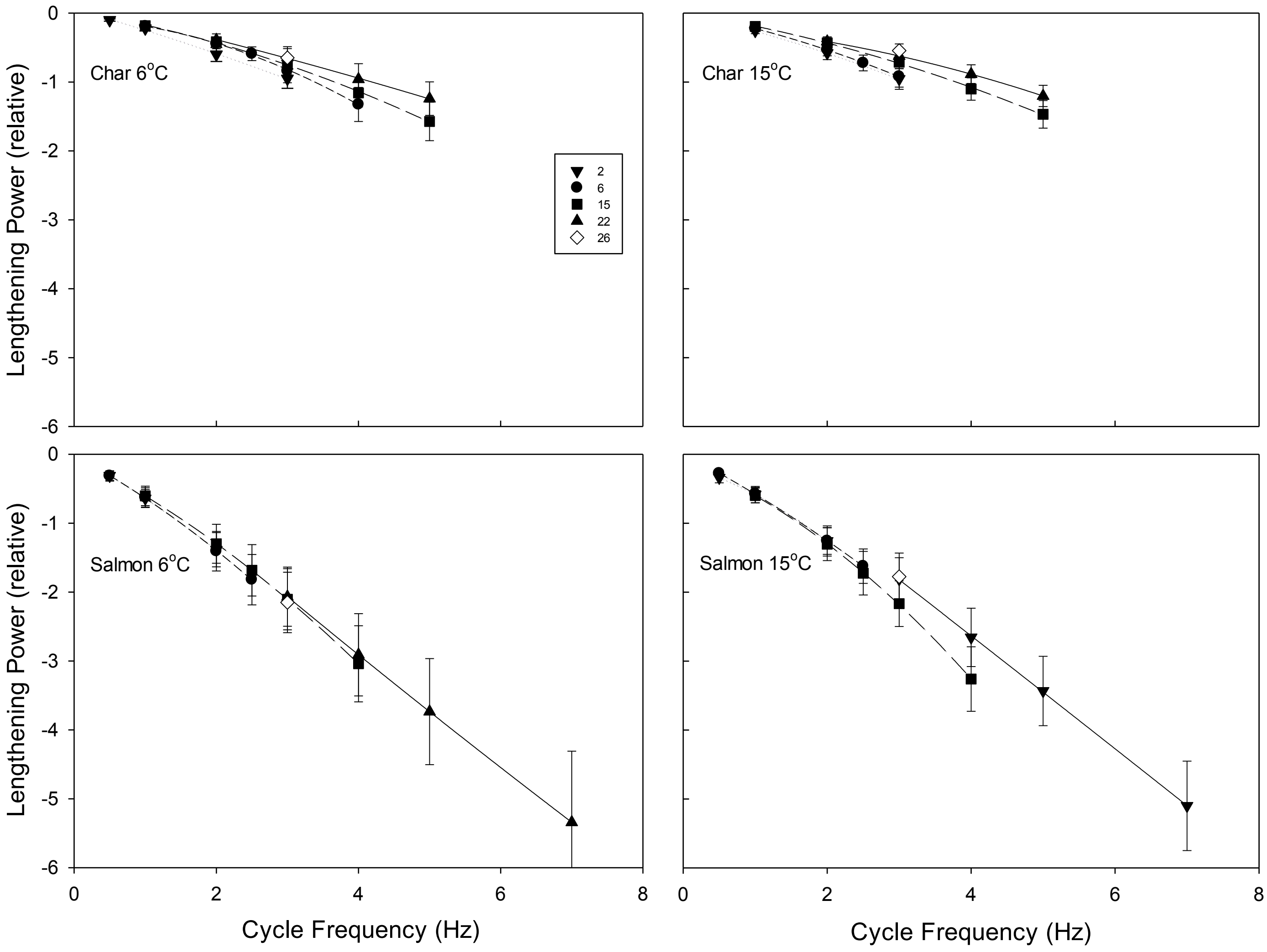

Fig. S3. Shortening power produced by ventricular muscle strips (trabeculae) from 6oC and 15oC acclimated Arctic char and Atlantic salmon at a range of test temperatures and contraction frequencies. Statistical tests were not performed since shortening power did not attain a maxima. See Fig. 3 for additional details.

Journal of Experimental Biology • Supplementary information

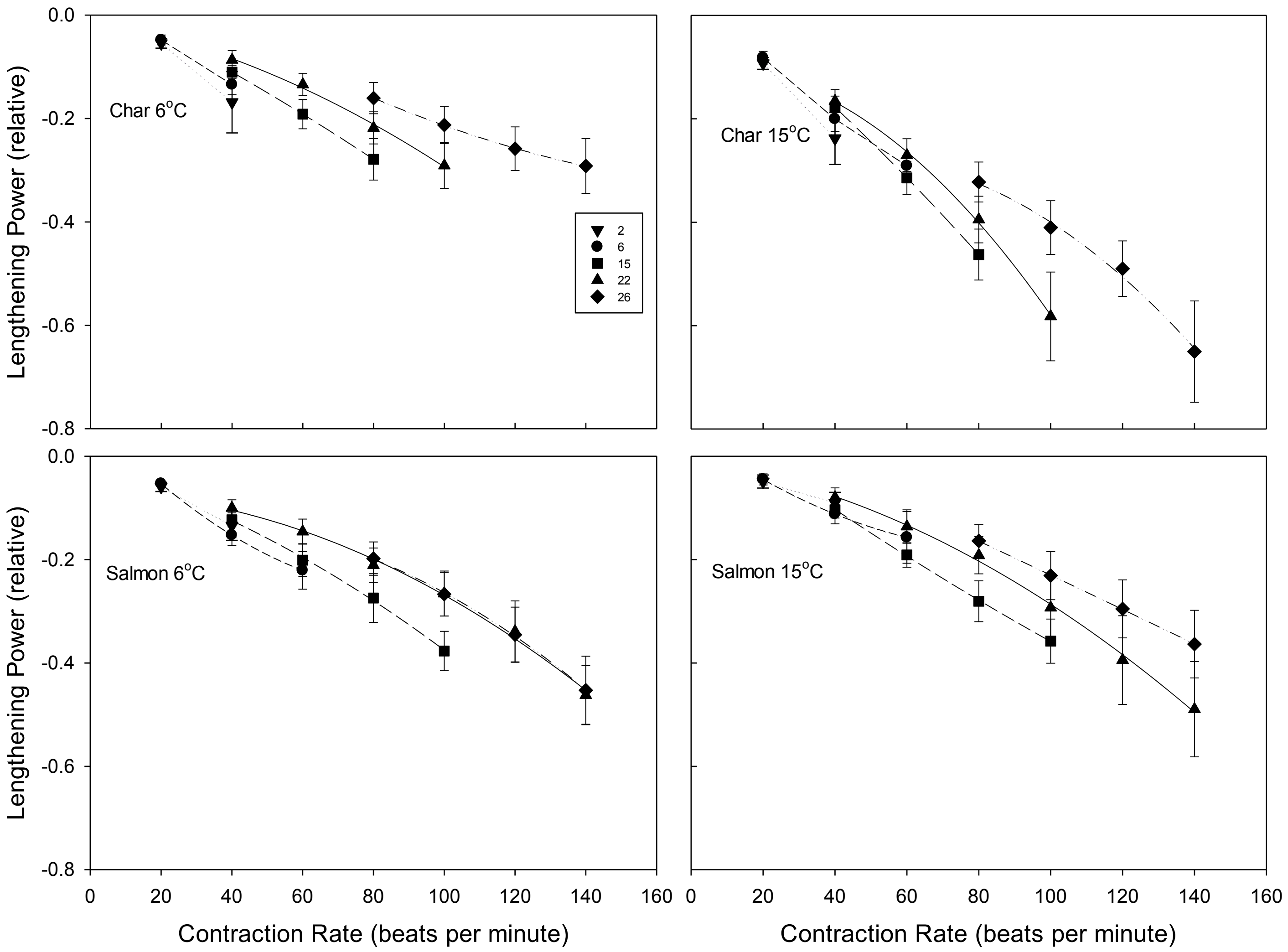

Fig. S4. Lengthening power (i.e., that required to stretch the muscle) of ventricular muscle strips (trabeculae) from 6°C and 15°C acclimated Arctic char and Atlantic salmon at a range of test temperatures and contraction frequencies. Statistical tests were not performed since lengthening power did not attain maxima. See Fig. 3 for additional details.

Journal of Experimental Biology • Supplementary information

Table S1. Parameters used to measure red skeletal muscle power. Values are means, showing the stimulus duration (in ms): stimulation phase (% of cycle). Standard deviations are omitted to reduce clutter, but were all within 5% or less of the mean.

Test Temp (°C)	Species / Acclimation Temp (°C)	Contraction Rate (Hz)							
		**0.5**	**1**	**2**	**2.5**	**3**	**4**	**5**	**7**
**2**	**Char/6**	983 : 10	475 : 3.8	206 : -6.9		108: -10			
	**Salmon/6**	881 : 10	431 : 2.5	170 : -3.1					
	**Char/15**		533 : 0	206 : -6.7		118 : -8.9			
	**Salmon/15**	1000 : 10	513 : - 2.5	218 : -9.4					
**6**	**Char/6**		475 : 10	186 : 1.3	136 : -1.3	115: -7.5	76 : -10		
	**Salmon/6**	944 : 15	406 : 10	184 : -1.3	143 : -5				
	**Char/15**		462 : 7.2	198 : -0.6	145 : -2.8	116 : -5.6			
	**Salmon/15**	884 : 17	519 : 4.4	227 : -3.3	165 : -6.7				
**15**	**Char/6**		500 : 14	254 : 5.0		151 : -0.6	110 : -6.3	86 : -8.1	
	**Salmon/6**		488 : 12	248 : 3.1	174 : 1.9	143 : -1.9	109 : -6.3		
	**Char/15**		500 : 15	243 : 4.4		144 : -0.6	102 : -4.4	74 : -7.8	
	**Salm/15**		521 : 10	249 : 1.5	199 : -3	163 : -6.5	121 : -12		
**22**	**Char/6**			251 : 11		183 : 2.5	128 : -0.8	103 : -3.8	
	**Salmon/6**					168 : 1.3	129 : -4.4	99 : -4.3	67 : -10
	**Char/15**			262 : 9.4		168 : 3.9	119 : 0	92 : -5	
	**Salm/15**					162 : 0.6	125 : -2.8	96 : -6.1	69 : -12
**26**	**Char/6**					204 : 0			
	**Salmon/6**					179 : 3.1			
	**Char/15**					180 : 4.4			
	**Salmon/15**					187 : 1.1			

Journal of Experimental Biology • Supplementary information

Table S2. Parameters used to measure ventricular muscle (trabecular) power. Values are means, showing the proportion of the strain cycle that comprised shortening (%): stimulation phase (% of cycle period, where 0% is equivalent to 0 degrees in a sine wave). Standard deviations are omitted to reduce clutter, but were all within 3.5% or less of the means.

Test Temp (°C)	Species / Acclimation Temp (°C)	Contraction Rate (beats min^{-1})						
		20	40	60	80	100	120	140
**2**	**Char/6**	44 : 6.9	60 : -8.3					
	**Salmon/6**	40 : 13	70 : -4					
	**Char/15**	40 : 4.4	70 : -6.7					
	**Salmon/15**	43 : 11	70 : -5.8					
**6**	**Char/6**	30 : 18	59 : -3.1					
	**Salmon/6**	30 : 22	69 : -2.5	72 : -4				
	**Char/15**	30 : 16	53 : -3.9	60 : 0				
	**Salmon/15**	30 : 19	52 : -0.5	80 : -10				
**15**	**Char/6**	20 : 33	33 : 13	58 : -0.63	69 : -6.4	70 : -7.5		
	**Salmon/6**	20 : 35	29 : 18	42 : 6.5	60 : -0.5	65 : -4.5	70 : -8.8	
	**Char/15**		40 : 12	60 : -1.1	66 : -5			
	**Salmon/15**		30 : 15	61 : -0.91	69 : -5	70 : -8.6		
**22**	**Char/6**		23 : 29	34 : 18	30 : 13	41 : 4.3	65 : -2.5	60 : -10
	**Salmon/6**		20 : 31	30 : 22	30 : 16	40 : 9.5	54 : 2	66 : -2.8
	**Char/15**		23 : 27	34 : 16	40 : 9.4	59 : -1.9	60 : -1	70 : -7.5
	**Salmon/15**		19 : 30	30 : 20	32 : 16	40 : 6.9	56 : 0	61 : -5.6
**26**	**Char/6**				29 : 21	30 : 16	40 : 11	40 : 6
	**Salmon/6**				30 : 25	30 : 20	40 : 12	40 : 6.1
	**Char/15**				30 : 19	39 : 13	40 : 10	40 : 2
	**Salmon/15**				30 : 23	30 : 19	40 : 13	50 : 6

Journal of Experimental Biology • Supplementary information

Table S3. Parameters used to measure ventricular muscle (trabecular) efficiency at different temperatures and rates of contraction. Phase is the time of onset of stimulation relative to the imposed strain cycle, expressed as a % of the cycle period, where 0% is equivalent to 0 degrees in a sine wave, and Short is the proportion of the strain cycle that comprised shortening, the remainder being lengthening. Temperatures indicate test temperature, and f_H is the contraction frequency. All fish were acclimated to 15ºC. Values are means (SEM).

	**15ºC**		**20ºC**				**25ºC**			
	**f_H 70 min^{-1}**		**f_H 70 min^{-1}**		**f_H 100 min^{-1}**		**f_H 70 min^{-1}**		**f_H 120 min^{-1}**	
	**Phase (%)**	**Short (%)**	**Phase (%)**	**Short (%)**	**Phase (%)**	**Short (%)**	**Phase (%)**	**Short (%)**	**Phase (%)**	**Short (%)**
**Char N=8**	-6.43 (0.854)	60.0 (0)	13.6 (0.854)	40.0 (0)	-5.00 (1.01)	60.0 (0)	20.0 (0)	30.0 (0)	12.9 (1.38)	40.0 (0)
**Salmon N=9**	-5.00 (0.786)	62.2 (1.39)	14.4 (0.944)	32.2 (1.39)	1.67 (1.36)	53.3 (2.72)	23.9 (0.693)	28.9 (1.05)	5.56 (0.944)	40.0 (0)

Journal of Experimental Biology • Supplementary information
